# Charge Storage Properties of Nanostructured Poly (3,4–ethylenedioxythiophene) Electrodes Revealed by Advanced Electrogravimetry

**DOI:** 10.3390/nano9070962

**Published:** 2019-07-01

**Authors:** Tao Lé, David Aradilla, Gérard Bidan, Florence Billon, Catherine Debiemme-Chouvy, Hubert Perrot, Ozlem Sel

**Affiliations:** 1 Laboratoire Interfaces et Systèmes Électrochimiques, CNRS, Sorbonne Université, LISE, UMR 8235, 75005 Paris, France; 2 CEA, INAC-SyMMES, CNRS, University Grenoble Alpes, F-38000 Grenoble, France; 3 Institute of Inorganic Chemistry, University of Goettingen, Tammannstrasse 4, 37077 Goettingen, Germany

**Keywords:** PEDOT, nanowires, (pseudo)-supercapacitors, charge storage mechanism, interfacial ion transfer, EQCM, AC–EG

## Abstract

PEDOT nanowires (NWs) directly grown on the conducting electrode of quartz resonators enable an advanced electrogravimetric analysis of their charge storage behavior. Electrochemical quartz crystal microbalance (EQCM) and its coupling with electrochemical impedance spectroscopy (*ac*–electrogravimetry or AC–EG) were used complementarily and reveal that TBA^+^, BF_4_^−^ and ACN participate in the charge compensation process with different kinetics and quantity. BF_4_^−^ anions were dominant in terms of concentration over TBA^+^ cations and the anion transfer results in the exclusion of the solvent molecules. TBA^+^ concentration variation in the electrode was small compared to that of the BF_4_^−^ counterpart. However, M_w_ of TBA^+^ is much higher than BF_4_^−^ (242.3 vs. 86.6 g·mol^−1^). Thus, TBA^+^ cations’ gravimetric contribution to the EQCM response was more significant than that of BF_4_^−^. Additional contribution of ACN with an opposite flux direction compared with BF_4_^−^, led to a net mass gain/lost during a negative/positive potential scan, masking partially the anion response. Such subtleties of the interfacial ion transfer processes were disentangled due to the complementarity of the EQCM and AC–EG methodologies, which were applied here for the characterization of electrochemical processes at the PEDOT NW electrode/organic electrolyte interface.

## 1. Introduction

Among the vast possibilities of electrode materials for pseudo-capacitors, electroactive conducting polymers (ECPs) are a very attractive solution due to their low price, non-toxicity and tunable chemical, electrical and physical properties [1]. These polymers can be electrochemically generated/doped in the presence of an electrolyte to obtain very good electrical conductivity (10 to 100 S·cm^−1^) and a wide electrochemical window.

Through the doping process, charges are transferred to the polymer chains with an excess of electrons in the case of n–doping and a lack of electrons in the case of p–doping. Then, ions from the electrolyte are inserted within the polymer matrix to maintain electrical neutrality. As the conducting polymer electrode is charged and discharged, the ionic exchanges with the electrolyte should happen very reversibly without damaging the polymer structure and should persist during a long cycling period, leading to suitable material for pseudo-capacitors. The most common ECPs used in literature are poly(pyrrole) (PPy), poly(aniline) (PANi), poly(thiophene) (PTh) and their derivatives such as poly(3,4–ethylenedioxythiophene) (PEDOT) [2,3,4]. Even though the specific capacitance values achieved with these materials (300–800 F·g^−1^) are lower than those obtained with certain transition metal oxides [5,6], extensive works have been made and reported in the past decades to investigate their potential for capacitor applications [1,2,3].

Despite the excellent electrochemical performances, one of the most important drawbacks of ECPs relies on their low cycling stability. Nanostructuration of ECPs represents a very promising strategy to overcome this hurdle through, as an example, the elaboration of nanocomposites made of ECPs and carbonaceous structures should be noted. More specifically, the great interest of ECP nanostructuration is also focused on one hand on the large pseudo-capacitance properties associated to their faradaic reactions and on the other hand on the large electrochemical active surface with an optimal ion diffusion path in the ordered nanostructure. According to the literature, various approaches have been already reported in this direction such as nanowires (NWs; PANi or PPy but also metallic NWs [7]), nanotubes (PEDOT) or nanofibers [8,9,10,11,12,13]. Among them, ECP-based nanowire (e.g., PANi) exhibited outstanding electrochemical performances in terms of cycling stability in ionic liquid electrolytes (500 galvanostatic charge–discharge (GCD) cycles with a capacitance retention of 92%) [14]. More recently, PEDOT NWs were also employed in the field of micro-supercapacitors. The PEDOT NW film electrode exhibited excellent electrochemical performances such as a high areal capacitance value of 667.5 mF cm^−2^ at 1 mA cm^−2^ and a capacitance retention of 94.3% after 10,000 GCD cycles [15], demonstrating the enormous potential of such nanostructures [2,14,16,17,18,19] in the field of supercapacitors.

Since the morphology of the ECP nanostructures has a crucial influence to enhance ion transfers at the electrode/electrolyte interface and thus on their electrochemical properties, finding a convenient and high-efficiency synthesis method with suitable nanostructure is required. This nanostructuration can be achieved in various ways: (i) Covering an existing nanostructure [20], (ii) synthesizing conducting polymer nanostructures using a template [15,18,21] or (iii) performing templateless growth of ECP NWs [11,22,23].

Among the synthesis methods, template-free electropolymerization of ECP NW electrodes is becoming a mature process with many recent developments reported in literature [11,13,24,25]. In the case of Py, the presence of only weak-acid anions such as HPO_4_^2−^ in the monomer solution during potentiostatic electrooxidation leads to a 10–20 nm thin non-conducting overoxidized polypyrrole (OPPy) film [26]. It was demonstrated that this film could be used to obtain a one-step nanowire growth process: When non-acidic anions are added to the solution in the presence of HPO_4_^2−^, PPy NWs grow surrounded by OPPy film. More specifically, the trick consists in using an amphoteric species like hydrogenophosphate or hydrogenocarbonate in the presence of a classical electrolyte. The basic form of the former consumes the protons released during the electropolymerization so that polymerization stops at early stage. As a constant potential is applied at the working electrode, water oxidation takes the relay and produces hydroxyl radicals and oxygen gas bubbles. The hydroxyl radicals overoxidize the already deposited PPy, except where oxygen nanobubbles are produced. These O_2_ nanobubbles that protect the PPy film against hydroxyl radicals i.e., against overoxidation allows the electropolymerization of Py to continue at these places leading to PPy nanowires [11,13,22,23,27]. A similar methodology is applied in this work for the PEDOT NW growth. To characterize the charge storage properties both electrochemically and gravimetrically, PEDOT NWs were directly deposited on the surface of conducting electrodes of quartz resonators. Electrochemical quartz crystal microbalance (EQCM) [28,29] and its coupling with electrochemical impedance spectroscopy (AC–EG) [30,31,32,33,34] were used complementarily to unravel the species participating in charge storage process in TBABF_4_ salt containing acetonitrile electrolyte. Due to the coupling with impedance spectroscopy, AC–EG has the ability to provide complementary information on the interfacial processes. Besides identifying each species participating in the charge compensation process, it can provide a quantitative picture together with the kinetics of transfer of each species, thereby providing a kinetic and gravimetric deconvolution [32,34]. Here, it is applied for the first time to scrutinize the electrochemical processes involved at the PEDOT NW electrode/organic electrolyte interface.

These nanostructured PEDOT electrodes are of significant interest not only in energy storage (i.e., pseudo-capacitors) [1,10] but also in organic–inorganic [35] or polymer-based optoelectronic devices such as polymer light-emitting diodes (PLEDs), as well as polymer solar cells (PSCs) [36].

## 2. Materials and Methods 

Electrode preparation and morphological characterization. Nanostructured PEDOT films were electrochemically prepared on the gold electrode (0.2 cm^2^) of quartz resonators (9 MHz, AWS, Spain) from an aqueous solution (bidistilled water) containing $0.2{\;M\;K}_{2}{\mathrm{HPO}}_{4}+2.10^{-2}{\;M\;\mathrm{LiClO}}_{4}+2.10^{-2\;}M\;3,4\text{–}\mathrm{Ethylenedioxythiophene}\;\left(\mathrm{EDOT}\right)$ in a three–electrode configuration applying 1.1 V vs. saturated calomel electrode (SCE) for 900 s. This optimal potential was considered according to previous studies regarding the electropolymerization of EDOT in aqueous solution [37]. A Pt grid was used as a counter electrode. The electropolymerization was conducted using a multichannel VSP3 potentiostat/galvanostat with Ec–Lab software (Biologic, Grenoble, France). All chemicals were purchased from Sigma Aldrich. The morphology of the PEDOT films were examined by using a ZEISS Ultra 55 scanning electron microscope equipped with energy dispersive X-ray spectrometry (EDX) element mapping analysis at an accelerating voltage of 10 kV.

Electrogravimetric characterization and evaluation of capacitive properties. EQCM measurements were performed in a solution of acetonitrile (ACN) containing 0.5 M tetrabutylammonium tetrafluoroborate (TBABF_4_), with a platinum grid as counter electrode and an acetonitrile-based Ag/Ag^+^ as reference electrode. The resonant frequency change, ∆*f*, of the quartz crystal was then converted into mass changes, ∆*m*, using the Sauerbrey equation ($\Delta f=-k_{s}\times \Delta m$ where *k_s_* is the experimental calibration constant ($16.3\times 10^{7}\;Hz\cdot g^{-1}\cdot cm^{2}$) [38]). An Agilent 4294A impedance analyzer was used to perform the electroacoustic impedance measurements [34] in order to validate gravimetric measurements. Mass per electron values, *MPE* = F × (Δ*m*/Δ*q*) were calculated from the EQCM data where F is the Faraday’s constant, ∆*m* and ∆*q* were obtained from the QCM and the CV data, determined from the oxidation or reduction scan directions [39].

For AC–EG [30], a four–channel frequency response analyzer (FRA, Solartron 1254) and a lab-made potentiostat (SOTELEM–PGSTAT) were used. The QCM was used under dynamic regime, the working electrode (WE) was polarized at a selected potential, and a sinusoidal small amplitude potential perturbation was superimposed. The ∆*f* corresponding to the mass response, ∆*m*, of the WE was measured simultaneously with the *ac* response, ∆*I*, of the electrochemical system. The frequency range was between 63 kHz and 10 mHz. The resulting signals were sent to a four-channel FRA, which allowed the electrogravimetric transfer function (TF) ∆*m/*∆*E(ω)*, and the electrochemical impedance ∆*E/*∆*I(ω)*, to be simultaneously obtained. 

The specific capacitance (*Cs*) was calculated using the following equation: *Cs* = *Q*/(Δ*V* × *m*), where *Q* is the average voltammetric charge, which is determined by integrating either the oxidation or reduction current of the corresponding CV curve, Δ*V* is the potential range, *m* is the active mass of the electrode. A total PEDOT NW mass of 28·μg cm^−2^ was estimated from QCM measurements. The areal capacitance was calculated taking into account the geometric surface of the electrode (A: 0.2 cm^2^) from the previous equation, where *m* is replaced by *A*.

## 3. Results and Discussion

**Figure 1:**
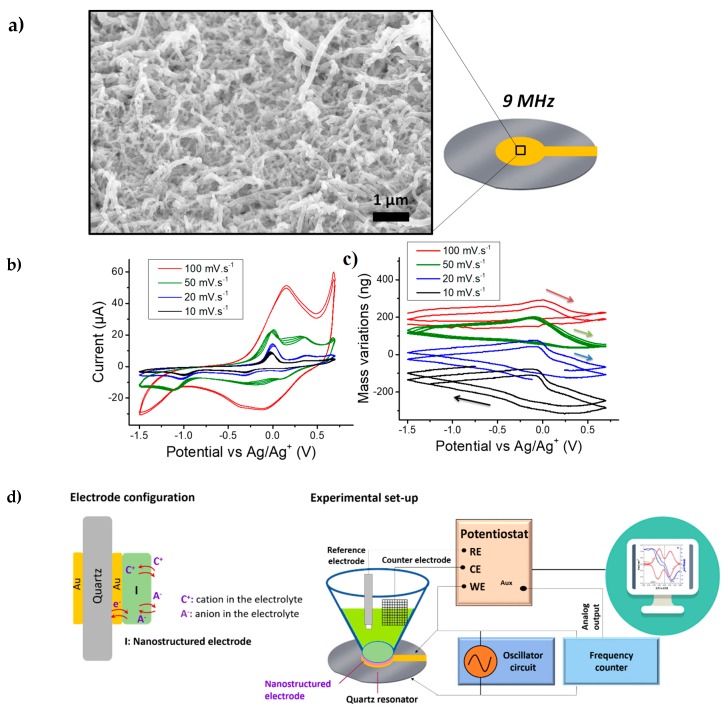
(**a**) SEM micrograph of PEDOT NWs on the surface of a 9 MHz quartz crystal microbalance (QCM); (**b**) cyclic voltammetry (CV) measurements on PEDOT NWs in ACN + 0.5M TBABF_4_ at various scan rates, (**c**) the corresponding mass variations obtained with QCM coupling to CV (two cycles) and (**d**) schematic presentation of the quartz configuration and experimental set-up used for electrochemical quartz crystal microbalance (EQCM).

Figure 1a depicts a SEM micrograph showing the morphology of the resulting nanowires. Under the electropolymerization conditions of this study, PEDOT nanowires with a diameter around 100 nm and a length of 1–2 µm were obtained. It is noted that the NWs are not completely perpendicular to the substrate but they exhibit a rather intertwined structure that fully covers the gold electrode of the quartz resonator. 

The electrogravimetric behavior was studied in a solution of ACN containing 0.5 M TBABF_4_. It is important to note that the gravimetric investigation of the nanostructured electrode can be influenced by the hydrodynamic damping in the presence of the electrolyte (i.e., the viscous drag force exerted on the electrode nanostructures causes a shift and a damping of the resonance frequency peak of the QCM) [29,34]. Hence, prior to the coupled QCM based studies, it is important to quantify this damping. The dissipation factor, $D=\frac{W}{f_{0}}$, where *W* is the full-width at half-height of the resonance peak and $f_{0}$ is the resonance frequency, has been proposed by Levi et al. to quantify the hydrodynamic damping of a QCM sensor [29,40,41,42]. The quality factor, $Q=\frac{f_{0}}{W},$ which is the inverse of dissipation factor can equally be used to quantify the degradation of the resonator’s quality. Accordingly, to calculate *Q*, electroacoustic admittance measurements were performed. PEDOT modified quartz resonators were investigated in air and ACN electrolyte (Table 1). A high quality factor, 2243 in ACN with nanostructured PEDOT film, allowed the use of these modified quartz resonators for gravimetric measurements in ACN-based electrolytes.

After this verification step, EQCM measurements were conducted from −1.5 V to 0.7 V vs. Ag/Ag^+^ in 0.5 M TBABF_4_ in ACN. This media has the advantage of possessing a wide electrochemical window compared to aqueous electrolytes (Figure 1b). An important resonant frequency increase (mass decrease) was observed up to one hour after electrolyte introduction in the electrochemical cell. It indicates a mass-loss inducing mechanism, which can be attributed to the exchange of the anions initially present in the PEDOT film (HPO_4_^2−^ or ClO_4_^−^ or both) with those of the testing solution (BF_4_^−^). Previous X-ray photoelectron spectroscopy (XPS) studies on PPy NWs prepared with a similar method evidenced 96 at% of Cl and 4 at% of P presence, indicating the ClO_4_^−^ as the main dopant [27,43]. Since the same synthesis methodology was applied here, except higher oxidation potential of EDOT over Py monomer, similar dopant composition could be predicted. Therefore, it can be assumed that the exchange of ClO_4_^−^ (Mw: 99 g·mol^−1^) with BF_4_^−^ (Mw: 86 g·mol^−1^) can lead to a mass decrease during the stabilization of PEDOT films in TBABF_4_ containing ACN.

After this stabilization step, EQCM measurements were performed on PEDOT NWs at different scan rates: 100, 50, 20 and 10 m·Vs^−1^ (Figure 1b, 1c and Figure 1d for the experimental set-up). The shape of the CV curves shown in Figure 1b is similar to previously reported results on PEDOT in ACN with LiClO_4_ and TBAClO_4_ [44]. A large residual current is observed after the oxidation wave, associated with pseudo-capacitive behavior [45], which is especially seen at 50 mV·s^−1^ and below. A *Cs* value of 25 F·g^−1^ (0.715 mF cm^−2^) was obtained at a scan rate of 100 mV·s^−1^. This value was found similar to other PEDOT-based nanowire system (e.g., PEDOT-coated silicon nanowires, *Cs*: 32 F·g^−1^) [46]. Periodic mass variations of around 175 ng were observed, with the mass increasing during reduction and decreasing rapidly just after the oxidation peak with a remarkable hysteresis (Figure 1c). At a first glance, this could be associated to a typical response of cations’ insertion during reduction and their expulsion during oxidation. 

The *MPE* calculated from the EQCM data could provide indications of the nature of the transferred species during cycling [20]. If only one species was involved in the charge storage process, the value of *MPE* would be its molar mass. The cation and anion contributions lead to negative and positive signs of *MPE* values, respectively [20]. A mean *MPE* value of −70 g·mol^−1^ was calculated right after the oxidation peak for potential from 0 V to 0.7 V vs. Ag/Ag^+^ (calculated from the EQCM data at 50 mV·s^−1^). This value was decreased down to around −10 g·mol^−1^ during the reduction process. The negative sign of the *MPE* corresponded to a predominant exchange of cations, but these values were lower than the mass of TBA^+^ cations (242.46 g·mol^−1^).

Classically, charge compensation during the doping/de-doping of ECPs occurs with the insertion/de-insertion of the anions. In certain cases, the anions are trapped in ECPs and neutralized by the cations. In the latter case, during the doping (oxidation), these cations can be expulsed for charge compensation purposes. Our EQCM results did not provide a clear indication of whether solely anions (BF_4_^−^) or cations (TBA^+^) intervene during the doping/de-doping of PEDOT NWs but rather indicate a “mixed behavior”. Different scenarios could explain this result: (i) BF_4_^−^ anions were expulsed at the same time as TBA^+^ cations were inserted, leading to a partial cancellation of the observed mass variations or (ii) an indirect participation of the solvent molecules to the charge compensation process. It is also noted that there was a slight change of the total mass variation, as a function of scan rate, (i.e., slightly higher *m*, at 10 mV·s^−1^), which may support the idea of multi-species contribution to the charge compensation with different kinetics. To unveil the mechanisms occurring in this pseudo-capacitive material and separate the contributions of the exchanged species, AC–EG was proposed to complement the EQCM data.

**Figure 2:**
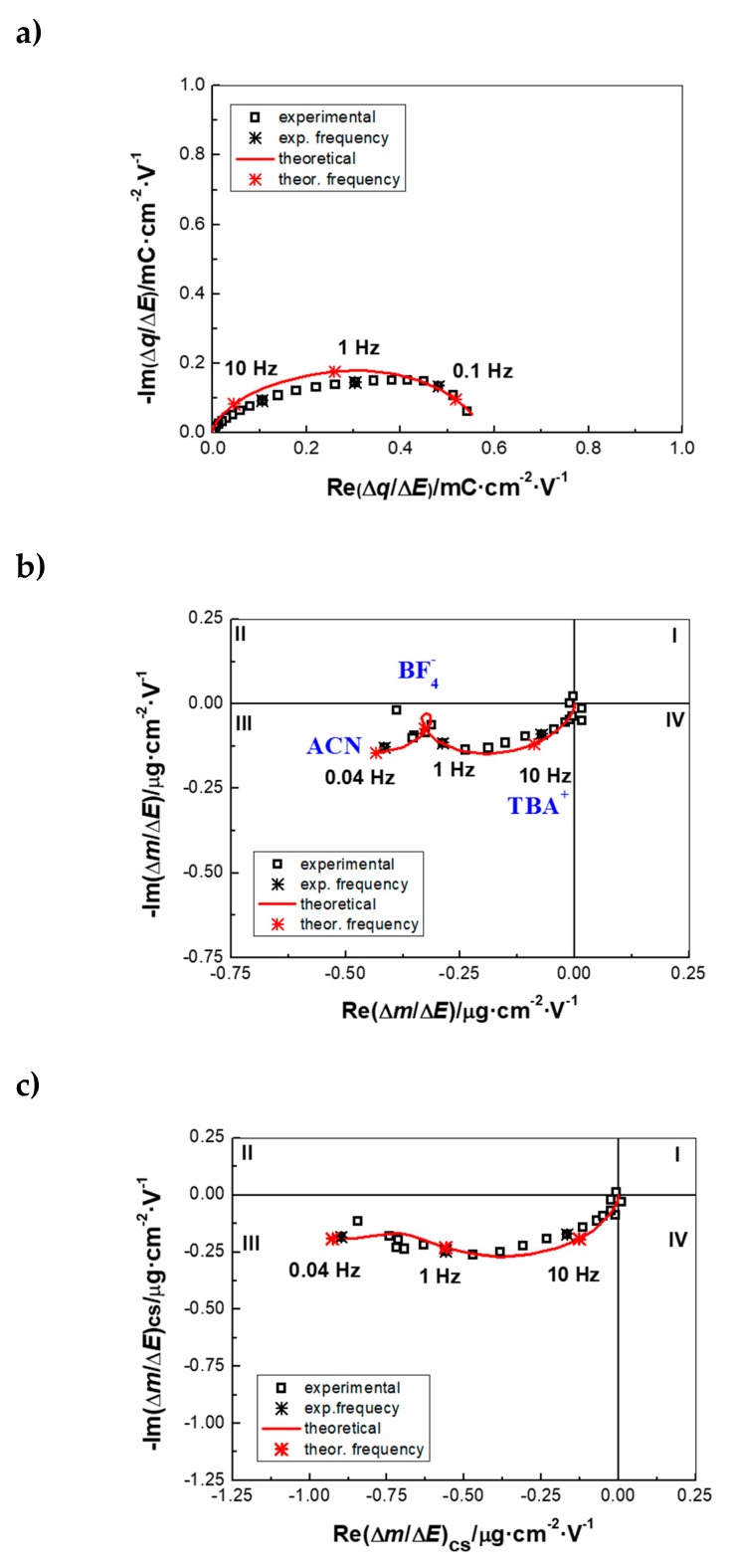
*Ac*-electrogravimetric data (experimental and fit) of PEDOT NWs in 0.5 M TBABF_4_ in ACN at 0.4V vs. Ag/Ag^+^: (**a**) Charge/potential transfer functions (TF), (**b**) mass/potential TF and (**c**) partial mass/potential TF only for cation and solvent. Fitted parameters: *K_c_:* 4.634 × 0^−4^, *G_c_:* 2.085 × 10^−8^*; K_a_:* 1.178 × 10^−4^, *G_a_:* −8.777 × 10^−8^; *K_s_:* 1.492 × 10^−5^, *G_s_:* −2.91 × 10^−9^ (c: TBA^+^, a: BF_4_^−^ and s: ACN). AC–EG is a coupling of QCM with electrochemical impedance spectroscopy (EIS), in lieu of cyclic voltammetry (CV) in the EQCM.

AC–EG measurements were performed on PEDOT NWs in ACN solution containing 0.5 M TBABF_4_ in a frequency range from 63 kHz to 0.01 Hz and polarization potentials ranging from −1.4 V to 0.6 V vs. Ag/Ag^+^, with one measurement every 0.2 V. Two important transfer functions (TF), charge/potential TF (Δ*q/*Δ*E*(*ω*)); derived from impedance, (Δ*E/*Δ*I*(*ω*)) and mass/potential TF (Δ*m/*Δ*E*(*ω*)) were obtained from AC–EG, as described earlier [30,31]. Figure 2 shows an example of *ac*-electrogravimetric data obtained at 0.4V vs. Ag/Ag^+^. The charge/potential TF, Δ*q/*Δ*E*(*ω*) presents one large depressed semi-circle, indicating interfacial transfer of multiple ions (Figure 2a). The time constants of participating species are probably not different enough to be seen as well-defined separate loops. The following equation is used to fit the charge/potential TF:

(1) $$\frac{\Delta q}{\Delta E}(\omega )=Fd_{f}\sum_{i}\frac{G_{i}}{j\omega d_{f}+K_{i}}\;(i:\;\mathrm{Ions}),$$

where *K_i_* represents the kinetics and *G_i_* describes the ease/difficulty of each species’ transfer at the electrode/electrolyte interface (*d_f_* is the film thickness and *F* is the Faraday’ constant). This fitting process indeed revealed the involvement of two different ionic species, which resulted in a good agreement between the experimental and the theoretical curves, without the possibility of their identification (Figure 2a) through the estimation of their molar weight based on AC–EG results. 

To do so, the mass/potential TF, Δ*m/*Δ*E*(*ω*), was analyzed (Figure 2b), which showed a complex behavior in agreement with the Δ*q/*Δ*E*(*ω*) TF in Figure 2a. The overall Δ*m/*Δ*E*(*ω*) curve enveloped the transfer of different species in terms of quantity (as well as in terms of frequency domain where each processes occur). Two parameters (*K_i_* and *G_i_*) previously obtained from the charge/potential TF for each ionic species were used for the fitting of the Δ*m/*Δ*E*(*ω*) response with the equation below [32]:

(2) $$\frac{\Delta m}{\Delta E}(\omega )=-d_{f}\sum_{i}M_{i}\frac{G_{i}}{j\omega d_{f}+K_{i}}\;(i:\;\mathrm{Ions}\;\mathrm{and}\;\mathrm{neutral}\;\mathrm{species}).$$

Due to the gravimetric aspect of this coupled method, uncharged species like solvent molecules could also be detected and identified by their molar mass (*M_i_*, Equation (2)). The fitting procedure revealed a configuration involving the transfer of cations (TBA^+^), anions (BF_4_^−^) and solvent (ACN) molecules, which leads to a good agreement between experimental and theoretical data (Figure 2b). This configuration is verified by partial TFs, for example by removing the anions (BF_4_^−^) contribution and analyzing the residual response, which also shows a good agreement of the experimental/theoretical data (Figure 2c). The mixed transfer of cations and anions was previously seen for PEDOT films, in TBAPF_6_ containing ACN electrolytes [47]. These processes depend on different parameters as the synthesis conditions of the ECPs, the nature of the dopant and the electrolyte ions [48,49,50]. Due to the impedance coupling in AC–EG, the kinetics of the interfacial transfer is also determined. It is noted that TBA^+^ appears at higher frequency domain than anions (BF_4_^−^), which are followed by free solvent (ACN) molecules. The latter have an opposite flux direction with BF_4_^−^, which may indicate that they are excluded from the PEDOT after the anions’ transfer (Figure 2b).

AC–EG results in the potential range of −1.4 V to 0.6 V vs. Ag/Ag^+^ show the persistence of this multi-species contribution, which is particularly clear in the range of 0 V to 0.6 V. This is in agreement with a relatively more reversible mass response in the reduction and oxidation direction at more anodic potentials of the EQCM data in Figure 1c.

By using *K_i_* and *G_i_* parameters, the relative concentration variations of each species (*C_i_ − C*_0_) can be estimated with further calculation, (i.e., through the integration of Equation (3), leading to Equation (4)):

(3) $$\frac{\Delta C}{\Delta E}(\omega )=-\frac{G_{i}}{j\omega d_{f}+K_{i}},$$

(4) $${C_{i}-C_{0}=\int_{E_{0}}^{E_{i}}\frac{\Delta C_{i}}{\Delta E}(\omega )dE|}_{\omega \to 0}=\int_{E_{0}}^{E_{i}}\frac{-G_{i}}{K_{i}}dE.$$

**Figure 3:**
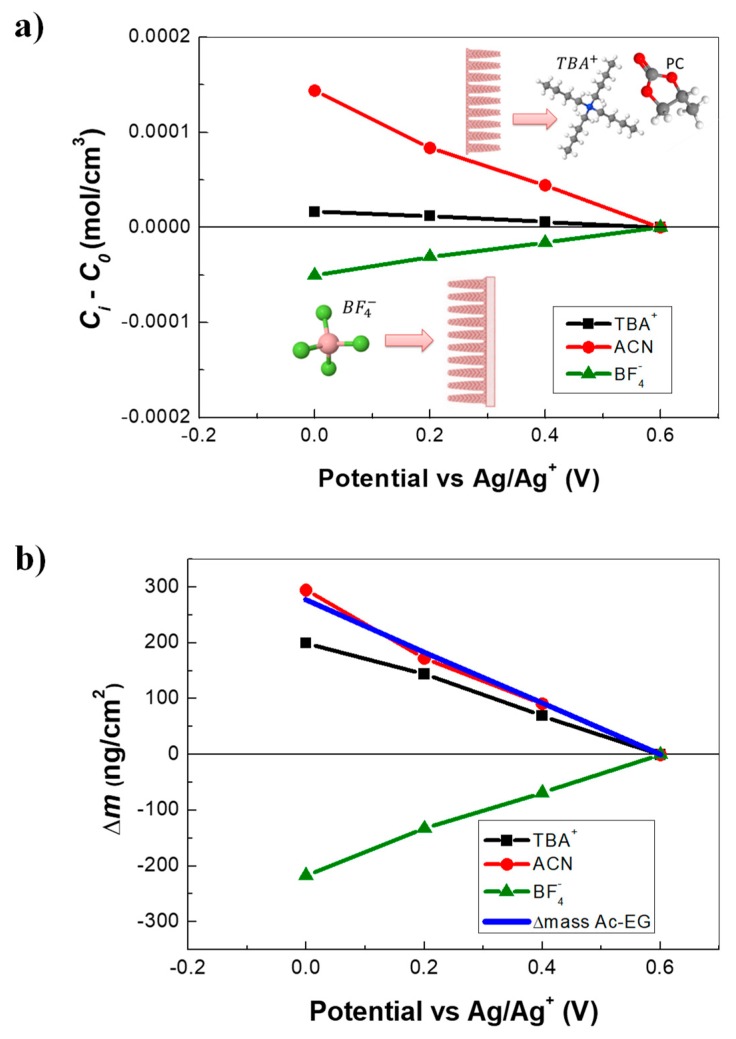
Complementary EQCM and *ac*–electrogravimetry (AC–EG) results of PEDOT NWs: (**a**) The variation of the relative concentration (*Ci − C*_0_) and (**b**) the relative mass variation of each species measured in ACN + 0.5M TBABF_4_. The reconstruction of total mass variation from AC–EG (in 0.6–0 V vs. Ag/Ag^+^ region).

The resulting concentration variations, depicted in Figure 3a, show that TBA^+^ cations were exchanged but in smaller quantities than BF_4_^−^. ACN contribution was quite significant compared with the ionic species in terms of concentration. As already explained, the solvent molecules were exchanged in the same flux direction with the TBA^+^ but in opposite flux direction with the BF_4_^−^. Since the solvent transfer occurs at low frequencies after the transfer of anions, the exclusion of the solvent molecules with the insertion of anions could be assumed, in agreement with previous reports in the literature, (i.e., considering *V*_ClO4_^−^ = 47Å^3^ and *V_CH3CN_* = 37Å^3^, one ClO_4_^−^ can replace approximately one acetonitrile molecule as a result of an exclusion effect [51]).

Using the concentration variations derived above, the mass variations induced by each species were calculated and shown in Figure 3b along with the sum of all contributions (reconstructed total mass from *ac*-electrogravimetry). The Δ*m_total_* obtained with *ac*–electrogravimetry (0.6 to 0 V vs. Ag/Ag^+^) was 280 ng·cm^−2^ (56 ng for 0.2 cm^−2^), which was in the same order of magnitude with the EQCM data obtained in the same potential range (Figure 2c, at 10 mV·s^−1^), without consideration of the large hysteresis in the EQCM response. This strengthens the validity of the multi-species contribution revealed by AC–EG. In spite of a small concentration variation, TBA^+^ cations were much heavier than BF_4_^−^ counterpart, thus TBA^+^ cations became significant, gravimetrically. Additional contribution of ACN with an opposite flux direction compared with BF_4_^−^, led to a net mass gain/lost during a negative/positive potential scan, masking the anions’ response. 

## 4. Conclusions

PEDOT NWs directly grown on the gold electrode of the quartz resonators enabled the electrogravimetric analysis of the charge storage behavior in TBABF_4_ containing ACN. Although the global EQCM mass response implies the major contribution of cations, the combination of the EQCM with AC–EG revealed TBA^+^, BF_4_^−^ and ACN participating, directly or indirectly, in the charge compensation process with different kinetics and quantity. BF_4_^−^ anions were dominant in terms of concentration over TBA^+^ and the anion transfer results in the exclusion of the solvent molecules. These results confirm that AC–EG is a very useful tool to operate a deconvolution of the EQCM measurements and unveil the ionic exchange mechanisms at the electrode/electrolyte interface.

## Figures and Tables

**Table 1:** Values obtained for the resonant frequency, peak width at half height, resistance (R) and quality factor (Q) for a quartz resonator with Au electrodes covered with PEDOT nanowires (NWs) in air and in ACN.

Configuration	Fluid Viscosity (*mPa.s*)	Fluid Density (*kg.m^−3^*)	Resonant Frequency (*MHz*)	Peak Width (*Hz*)	R (*Ω*)	Quality Factor
**PEDOT NW in air**	0.0184	1.17	8.983 (±10 Hz)	922 (±18)	67 (±1)	9734 (±195)
**PEDOT NW in ACN**	0.34	786	8.980 (±10 Hz)	4002 (±80)	273 (±5)	2243 (±45)
